# Potential of Wollastonite-Based Brushite Cement for the Conditioning of Radioactive Waste Contaminated by ^90^Sr

**DOI:** 10.3390/ma19061136

**Published:** 2026-03-14

**Authors:** Jihane Jdaini, Céline Cau Dit Coumes, Yves Barré, Marie-Noëlle de Noirfontaine, Mireille Courtial

**Affiliations:** 1 Commissariat à l’énergie atomique et aux énergies alternatives (CEA), DES, ISEC, DPME, SEME, University Montpellier, 30207 Bagnols-sur-Cèze, France; celine.cau-dit-coumes@cea.fr; 2 Laboratoire des Solides Irradiés, CEA, CNRS, Ecole Polytechnique, Institut Polytechnique de Paris, 91128 Palaiseau, France; marie-noelle.de-noirfontaine@polytechnique.edu (M.-N.d.N.); mireille.courtial@polytechnique.edu (M.C.); 3 Commissariat à l’énergie atomique et aux énergies alternatives (CEA), DES, ISEC, DMRC, STDC, University Montpellier, 30207 Bagnols-sur-Cèze, France; yves.barre@cea.fr; 4 Université d’Artois, 62408 Béthune, France

**Keywords:** radioactive waste, strontium, cement, leaching, gamma irradiation

## Abstract

This work investigates the potential of wollastonite-based brushite cement (WBC) for the stabilization and solidification of radioactive waste contaminated by ^90^Sr. This phosphate binder was formed by the reaction of wollastonite (CaSiO_3_) with a phosphoric acid solution containing borax and metallic cations (Al^3+^, Zn^2+^). Two cement pastes were investigated: a commercial binder (WBC-C) and an optimized formulation (WBC-O), produced using a zinc-free mixing solution with a higher aluminum content than that of WBC-C. Mineralogical characterizations using XRD, TGA, XRF, SEM-EDX, and Raman spectroscopy showed that both materials mainly contained amorphous hydrated silica and calcium aluminophosphate, along with crystalline brushite, residual wollastonite, and quartz. The stability of WBC-C under γ-irradiation was evaluated up to a dose of 1 MGy. The only observable effect was water radiolysis, leading to dihydrogen production at yields comparable to Portland cement matrices and geopolymers. Strontium leaching, assessed using the ANSI/ANS-16.1-2003 (R2008) procedure, followed a two-stage release mechanism combining surface wash-off and diffusion. The apparent diffusion coefficient D_a_ of Sr in WBC-C was markedly lower than typical values reported for Portland cement matrices. WBC-O exhibited enhanced Sr retention, possibly due to its higher aluminum content, which refines mesopores and reduces diffusion pathways accessible to Sr. WBC binders therefore appear to be promising candidates for strontium immobilization.

## 1. Introduction

Aqueous radioactive waste, primarily contaminated with cesium and strontium, may be generated by cleaning operations carried out as part of the decommissioning of old nuclear facilities [1]. After a volume-reduction, typically involving evaporation, co-precipitation and filtration, or sorption onto ion exchange materials, the resulting low-level or intermediate-level radioactive waste is usually encapsulated in a cement-based matrix prior to disposal [2]. This conditioning step is intended to prevent waste dispersion, facilitate handling and transportation, and limit the release of hazardous elements that could endanger human health and the environment. Portland cement, possibly blended with supplementary cementitious materials such as blastfurnace slag or fly ash, is commonly used to design cement-based matrices [3]. This binder is indeed readily available, compatible with aqueous waste (as the water in the waste is used up for cement hydration), and generally exhibits good mechanical strength after hydration, long-term stability, and high alkalinity—favorable to the precipitation and confinement of many radionuclides, including actinides [3,4]. Nonetheless, its capacity to retain critical radionuclides, such as ^137^Cs and ^90^Sr, may be limited [5,6,7]. This study focuses on ^90^Sr, a high-energy β-emitter that is particularly toxic due to its chemical similarity to calcium, which allows it to readily bind to bone tissues [8,9]. The aim of this work is to evaluate the potential of a calcium phosphate binder as an alternative to Portland cement for enhancing strontium retention. Calcium phosphate cements are used in dentistry and orthopedic bone-filling surgeries since they form products with chemical compositions similar to those of bones and teeth [10,11,12,13,14,15,16]. In particular, wollastonite-based brushite cement (WBC) is a phosphate binder prepared by mixing wollastonite, a natural meta-silicate mineral (CaSiO_3_), with a solution containing phosphoric acid, boron, and metallic cations (such as Al^3+^ and Zn^2+^) [17,18,19]. Wollastonite reacts through dissolution/precipitation, resulting in a multiphase material that includes brushite (CaHPO_4_·2H_2_O), amorphous calcium aluminophosphate, amorphous silica, and residual wollastonite [17]. Brushite (CaHPO_4_·2H_2_O) is well-known for its flexible structure, allowing for the substitution of strontium in place of calcium [20,21,22,23]. Additionally, as compared to other phosphate binders, WBC offers the advantage of being cost-effective due to the raw materials used and the ability to prepare it at room temperature.

The retention properties of solidified cement matrices used for the immobilization of radioactive waste are commonly assessed through leaching tests. In a previous study [24] where leaching experiments were conducted according to the ANS/ANSI-16.1 standard [25], we showed that strontium retention was significantly improved when using a commercial WBC binder compared to conventional Portland cement. The apparent diffusion coefficient (D_a_) of strontium in WBC paste was found to be approximately three orders of magnitude lower than values typically reported for Portland cement-based matrices. These findings highlight the strong potential of WBC-based materials for effective strontium encapsulation.

However, beyond radionuclide retention challenges, another concern for cement-based materials devoted to radioactive waste conditioning is the hydrogen gas production resulting from radiolysis of water—either present as free water in the pore solution, or chemically bound to the cement hydrates [26,27,28,29,30]. Gas accumulation in the cemented waste packages may induce pressure build-up, finally leading to cracking, which decreases their mechanical and confining properties [31,32]. In addition, hydrogen gas may form combustible or explosive mixtures with atmospheric oxygen when its concentration exceeds 4%, which raises a major safety issue for disposal facilities.

To fully assess the suitability of WBC-based materials for radioactive waste conditioning, it is important to investigate their behavior under ionizing radiation, as would occur in the presence of radionuclides within encapsulated waste. Although strontium is a 100% β-emitter, it may be conditioned with other radionuclides emitting α or βγ radiations, which justifies focusing on γ-irradiation in this study. Accordingly, the first part of this work evaluates the response of a commercial WBC binder, along with its main crystalline hydrate, brushite, to external γ-irradiation using a ^60^Co source. The total integrated dose (1–5 MGy) was representative of the dose that may be absorbed by a cement-waste form over its lifetime. The dihydrogen gas production was measured by gas chromatography, and possible structural damage in the solid phases was looked for using X-ray diffraction.

In addition to the behavior of WBC binders under irradiation, the influence of the formulation parameters on strontium retention also needs to be assessed, as the mineralogy of these binders depends on the composition of the mixing solution. Recent investigations by Laniesse et al. [18] examined how the concentrations of phosphoric acid, boron (added as borax or boric acid), and metallic cations (such as Al^3+^ and Zn^2+^) influence the setting time, mineralogy, and mechanical properties of WBC binders. Boron was found to delay the setting time of the cement paste and reduce its self-heating during hydration [18]. However, the resulting material exhibits poor mechanical properties. In contrast, metallic cations acted as setting accelerators. Specifically, zinc promoted the formation of scholzite (CaZn_2_(PO_4_)_2_·2H_2_O), a crystalline calcium zinc phosphate, while aluminum led to the formation of an amorphous calcium aluminophosphate [18]. Increasing the aluminum concentration in the mixing solution significantly improved the mechanical strength of the hardened material. Building on these results, Laniesse et al. [18] identified an optimal composition range for the mixing solution ([H_3_PO_4_] = 9 mol·L^−1^, [Al^3+^] = 1.8 to 2.5 mol·L^−1^, [B] = 0.2 to 0.6 mol·L^−1^) that produces a material with suitable properties for waste conditioning, including a setting time between 4 and 48 h, a compressive strength greater than 30 MPa, and a maximum heat flow below 5 mW/g.

While the effect of the mixing solution composition on the mineralogy and mechanical properties of WBC binders is well established, its influence on strontium retention remains unclear. To address this gap, the second part of this study investigates a WBC paste formulated with an optimized mixing solution ([H_3_PO_4_] = 9 mol·L^−1^, [Al^3+^] = 2.5 mol·L^−1^, [B] = 0.2 mol·L^−1^). Leaching tests, conducted in accordance with the ANS/ANSI-16.1 standard procedure [25], were used to quantify strontium release and to determine the corresponding apparent diffusion coefficient (Dₐ) and leachability index (LI). The results are compared with those previously obtained for a commercial WBC binder [24] and for conventional cement-based matrices.

## 2. Experimental

### 2.1. Material and Sample Preparation

To investigate the influence of WBC formulation on strontium retention, two cement pastes were studied in this work (Table 1). Both were prepared using the same commercial wollastonite powder (CaSiO_3_, supplied by Sulitec, Saint Alban de Roche, France), characterized by a specific surface area of 1.2 m^2^·g^−1^ (BET surface area measurement with nitrogen, Micromeritics ASAP 2020, Micromeritics Instrument Corporation, Norcross, GA, USA) and a particle size distribution of d_10_ = 2.8 µm, d_50_ = 15.2 µm, and d_90_ = 48.3 µm (measured by laser granulometry in ethanol, Mastersizer 3000, Malvern Panalytical, Malvern, UK). It was slightly carbonated, containing 2.1 ± 0.2 wt.% CaCO_3_, as determined by thermogravimetry analysis (TGA, Netzsch STA 409 PC LUXX, Netzsch, Selb, Germany), and contained trace amounts of quartz.

The first paste, referred to as WBC-C (commercial formulation), was prepared using a commercial mixing solution (also provided by Sulitec), with the following composition: [H_3_PO_4_] = 9.3 mol·L^−1^, [Al^3+^] = 1.6 mol·L^−1^, [Zn^2+^] = 1.5 mol·L^−1^, and [B] = 0.6 mol·L^−1^ (added as borax). The second paste, referred to as WBC-O (optimized formulation), was prepared using a synthetic mixing solution based on optimized compositions reported in [18], containing [H_3_PO_4_] = 9 mol·L^−1^, [Al^3+^] = 2.5 mol·L^−1^, and [B] = 0.2 mol·L^−1^. Sr-doped WBC-C and Sr-doped WBC-O pastes were also prepared, as summarized in Table 1.

All pastes were prepared using a standardized mixer in accordance with European standard 196-1. The two components were mixed for 5 min at low speed, with a liquid-to-solid (L/S) weight ratio of 1.25, resulting in a Ca/P molar ratio also equal to 1.25. For leaching experiments, stable strontium was used as a surrogate for its radionuclide and was added to the mixing solution as Sr(NO_3_)_2_ (Merck, Darmstadt, Germany, >99% purity) at a concentration of [Sr^2+^] = 1 g·L^−1^. Paste samples for leaching experiments were cast into polypropylene cylindrical containers (30 mL in volume) that were tightly sealed and cured for 28 d at room temperature. Those intended for irradiation tests were poured into 15 mL plastic centrifuge tubes (“SuperClear”, provided by VWR) that were immediately sealed to prevent desiccation and stored for 28 days at room temperature.

The WBC-C paste was used for both irradiation and leaching experiments. Synthetic brushite (CaHPO_4_·2H_2_O) was used in some irradiation experiments. The brushite powder (d_10_ = 3.0 µm, d_50_ = 11.8 µm, d_90_ = 27.8 µm, and specific surface area = 1.4 ± 0.1 m^2^·g^−1^) was provided by Accros Organics (Geel, Belgium). It contained 95.80 wt.% brushite, 2.05 wt.% monetite CaHPO_4_, and 2.15 wt.% newberyite MgHPO_4_·3H_2_O, as determined in [33].

### 2.2. Irradiation Experiments

The WBC-C samples used for irradiation experiments consisted of small cylinders (15 mm in diameter, approximately 5.6 cm in height) containing 10 mL of cement paste and cured for 28 d in their airtight plastic tubes at room temperature. Prior to irradiation, the samples were demolded, weighed (~20 ± 2 g), introduced into 100 mL glass tubes, deaerated by applying 3 cycles of depressurization at 30 hPa and pressurization with argon, and finally flame-sealed under 900 hPa of pure argon (Alphagaz 1 of Air Liquide, Saint-Denis, France). A previous study showed that water loss due to desiccation during the sealing process was less than 1% of the total water content of the paste samples [34].

Irradiation experiments were also carried out on brushite powder. Samples of 1 ± 0.05 g were introduced in 100 mL glass tubes that were deaerated and sealed following the same protocol as for the cement pastes.

Gamma irradiations were performed in a Steris Gammatec experimental irradiator located in Marcoule, France, and equipped with a ^60^Co source (energy of 1.2 MeV). The temperature of the irradiation chamber was regulated between 20 and 25 °C, and the dose rate was close to 1000 Gy·h^−1^. The integrated doses were equal to 100 kGy, 250 kGy, 500 kGy, and 1 MGy for the cement pastes, and to 250 kGy, 500 kGy, 750 kGy, 1 MGy, 2.5 MGy, and 5 MGy for brushite powder. The experiments were conducted in triplicate for pastes and in duplicate for powders.

In a recent study, Herin et al. [35] showed that hydrogen gas release from portlandite exposed to accelerated electrons or gamma irradiation occurs almost instantaneously for H_2_ formed near or at the surface of the crystals, but continues, at a slower rate, over a two-month period for H_2_ trapped within the bulk of the material. Based on these observations, the authors thus recommended heating the sample at 180 °C for 3 days to fully release the stored hydrogen. However, this thermal treatment was unsuitable for our samples, as it would cause significant thermal decomposition of the cement hydrates (see Section 3.1). Instead, the glass tubes were stored for three weeks after irradiation to allow hydrogen to diffuse through the cementitious materials.

Diffusion of dihydrogen through the pore network of the paste samples can be described by Fick’s second law. For small cylindrical samples (15 mm diameter), the characteristic diffusion time of H_2_ from the center to the surface can be approximated as:

(1) $$\tau \sim \frac{\varepsilon l^{2}}{D_{a}}$$

with *l* = 7.5 ± 10^−3^ m, *e* as the porosity, and *D_a_* as the H_2_ apparent diffusion coefficient. A 21 d diffusion time (corresponding to the delay between the end of irradiation and gas analysis) would require a diffusion coefficient of 8.10^−12^ m^2^·s^−1^. The WBC-C pore network had a water saturation degree assessed at 45 ± 18% after 28 d (Supplementary Information S1). Its apparent H_2_ diffusion coefficient was unknown. A rough estimate was obtained by comparison with data reported for a Portland cement paste (w/c = 0.35), having similar total porosity but finer pores. D_a_ ranged between 10^−6^ and 10^−7^ m^2^·s^−1^ for similar water saturation degrees [36]. Given the coarser pores in WBC-C (see Section 3.1), similar or higher D_a_ values may be expected, i.e., several orders of magnitude above 8 × 10^−12^ m^2^·s^−1^. The three-week delay between irradiation and gas analysis should thus be sufficient for H_2_ to diffuse to the headspace of the irradiation cell.

Subsequently, the dihydrogen content present in the gas phase was determined by gas chromatography (Agilent 7820A, Agilent Technologies, Santa Clara, CA, USA), using argon as the carrier gas, a capillary column, and a thermal conductivity detector. The H_2_ concentration (in mol·kg^−1^) was given by the following equation (Equation (2)):

(2) $$\left[H_{2}\right]=\frac{P\cdot f{(H}_{2})\cdot V_{t}}{R\cdot T\cdot m_{sample}}$$

where *P* (in Pa) is the gas pressure in the glass tube after irradiation, *f*(*H*_2_) is the volume fraction of H_2_ determined by gas chromatography, *V_t_* is the volume of the glass tube (in m^3^), *R* (in J.mol^−1^·K^−1^) is the ideal gas constant, *T* (in K) is the sample temperature, and *m_sample_* (in kg) is the sample mass. The radiolytic yield of hydrogen *G*(*H*_2_)*_material_* (in mol·J^−1^) was then given by the slope of the gas production versus absorbed dose *D* (in Gy) plot (Equation (3)):

(3) $${G\left(H_{2}\right)}_{material}=\frac{\left[H_{2}\right]}{D}$$

Therefore, *G*(*H*_2_)*_material_* represented the amount of hydrogen produced by the material for 1 J of absorbed energy. The experimental error associated with the determination of *G*(*H*_2_)*_material_* was close to 10% for radiolytic yields higher than 10^−9^ mol·J^−1^, but increased up to 50% for smaller yield values.

Since water was the only significant source of hydrogen in the irradiated samples, the hydrogen radiolytic yield *G*(*H*_2_)*_material_* was also standardized with respect to the total mass of water (*w_water_*) present in the samples (Equation (4)):

(4) $${G\left(H_{2}\right)}_{water}=\frac{{G\left(H_{2}\right)}_{material}}{w_{water}}$$

This normalized radiolytic yield was useful to compare the H_2_ production when the samples did not contain the same amount of water.

### 2.3. Leaching Experiments

**Figure 1:**
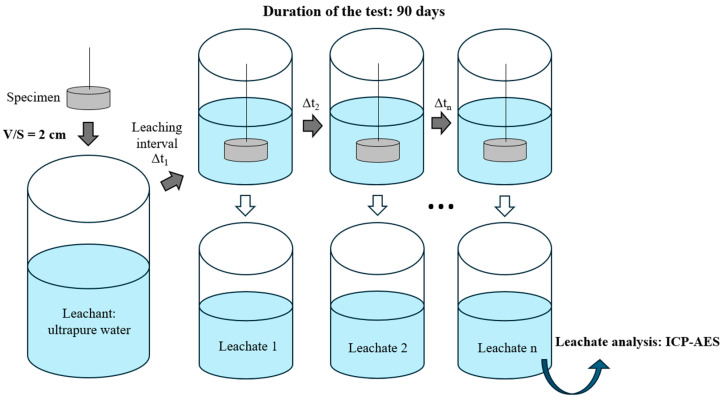
Leaching test procedure.

Leaching experiments were conducted following a protocol derived from the ANSI/ANS-16.1 specification [25]. The test was performed at room temperature (22 ± 2 °C) by immersing a cylindrical paste specimen with a diameter of 27.4 mm and a height of 16.2 mm for 90 d in ultrapure water as the leachant for 90 d. The leachate was refreshed with new water nine times over the course of 3 months (at 2 h, 7 h, 24 h, 48 h, 5 d, 7 d, 14 d, 45 d, and 90 d) to limit the accumulation of dissolved species (Figure 1). The volume of the leachant was set to 2 cm per unit of external geometric surface area of the specimen, rather than the recommended 10 cm. This reduction in leachant volume increased the concentration of strontium in the leachates, making it easier to detect. A previous attempt with the 10 cm ratio resulted in Sr concentrations below the detection limit (1 ppb) of the analytical method used. However, this change in the protocol also led to more significant changes in the leachant composition between renewals. Note that, from an application perspective, this lower V/S ratio is less removed from repository conditions, where cement-waste forms are expected to undergo progressive resaturation by a limited amount of water rather than leaching under conditions of water in large excess. Strontium ions, as well as the major elements of the cement matrix (Ca, P, Si, Al, and Zn) released in the leachates, were analyzed using inductively coupled plasma atomic emission spectroscopy (ICP-AES, ThermoFisher iCAP 3600 duo, Thermo Fisher Scientific, Waltham, MA, USA).

The cumulative fraction of strontium leached (*CFL*) was calculated following Equation (5):

(5) $$CFL=\frac{\sum A_{n}}{A_{0}}$$

where *A_n_* [g] is the amount of strontium released from the specimen during leaching interval n and *A*_0_ [g] is its initial amount in the specimen.

### 2.4. Solid Phase Characterization

#### 2.4.1. Hydration Stoppage

Before characterization, the pristine and irradiated cement pastes were submitted to hydration stoppage through solvent exchange with isopropanol following the procedure recommended by RILEM TC-238 SCM [37]. The residual isopropanol was then eliminated by gentle drying in a desiccator maintained at 20 °C and 23% R.H. to prevent any thermal degradation of brushite [38].

#### 2.4.2. X-Ray Diffraction

The pristine and irradiated paste samples were characterized by X-ray diffraction (XRD) using a Panalytical X’Pert Pro diffractometer (Malvern Panalytical, Almelo, Netherlands). First, the samples were finely ground by hand at a particle size below 80 µm. Since wollastonite crystallizes as fine needles [39], the paste samples were analyzed using the Debye–Scherer configuration (transmission mode). They were introduced in Lindeman tubes (Ф = 0.7 mm) and mounted on a rotating goniometric head during measurement to minimize the preferred orientation effect. Silicon (Alfa Aesar, Heysham, UK, 99.5% metal basis, ≈40 µm) was added to the samples as an internal standard (at a content of 10 wt.%) when the different phases were quantified with the Rietveld method [40]. The XRD data were recorded by using copper radiation (λ_CuK_ = 1.5418 Å) at room temperature in the 2-θ range 5–70° with a step size of 0.013° for a total counting time of 2 h. Diffrac.EVA software (Bruker-AXS; V4.3, 2010–2018) and PDF-2 database (Powder Diffraction File, version 2009) were used for phase identification. Structural refinement of the XRD pattern of pristine cement paste was based on the crystallographic structures of brushite, wollastonite (triclinic), and quartz (impurity in wollastonite), respectively, as established by Schofield et al. [41], Ohashi [42], and Antao et al. [43]. It was carried out with TOPAS software (Bruker-AXS; V6, 2016) using the fundamental parameters approach and the ICSD database (ICSD files n° 172258, 201537, and 162490 for brushite, wollastonite, and quartz, respectively). For all adjustments, the refined parameters were the phase scale factors, sample displacement, background modeled by a Chebyshev polynomial (order 5) combined with a 1/X term, unit cell, and microstructural parameters. The atomic positions, site occupancies, and temperature factors were kept constant during the refinement. Preferred orientation of brushite and wollastonite in the samples was taken into account using the March–Dollase correction [44].

Brushite samples were analyzed using a D8 Advance powder X-ray diffractometer (Bruker, Karlsruhe, Germany) with a copper radiation source in the Bragg–Brentano geometry (reflexion mode). The instrument was operated in step-scan mode, between 5° and 120° (2θ), with a 0.007° (2θ) step and 1 s per step. An automatic anti-scattering knife was used to reduce unwanted scattered radiation by the atmosphere at low angles from the main beam. In order to minimize preferential orientation, the powders were prepared on Si wafers as zero-background holders to improve the detection of a potential diffusion halo. The Rietveld refinements of brushite samples were performed following the method previously described.

#### 2.4.3. Raman Spectroscopy

Raman spectroscopy was performed on pristine and irradiated paste powders using a Horiba XploRA Plus (Horiba, Kyoto, Japan) equipped with a Syncerity detector cooled to −60 °C. Spectra were collected over 300–1600 cm^−1^ and 3300–3600 cm^−1^ with 10 accumulations of 10 s each. A 532 nm laser coupled with a 2400 g/mm diffraction grating was used, and the laser power was set to 100%.

#### 2.4.4. Thermogravimetry

To determine the water content of cement pastes and brushite submitted to irradiation, thermogravimetric analyses (TGA) were carried out on ground samples using a TGA/DSC Netzsch STA 409 PC instrument (Netzsch STA 409 PC LUXX, Netzsch, Selb, Germany) operating under nitrogen (gas flow set at 50 mL·min^−1^) at 10 °C·min^−1^ up to 1000 °C. The curves were corrected from buoyancy effects by performing a blank subtraction.

#### 2.4.5. X-Ray Fluorescence

X-ray fluorescence (XRF) measurements were carried out on the solid fraction of the pristine cement paste to determine its chemical composition. The ground powder was fused at 950 °C with a sample-to-lithium metaborate ratio equal to 0.05. The pellet was then analyzed using a Bruker XRF instrument (S8 Tiger, Karlsruhe, Germany).

#### 2.4.6. Scanning Electron Microscopy

Polished sections were prepared by vacuum impregnation of 28 d-old WBC pastes (after hydration stoppage) with epoxy resin. The samples were subsequently polished to a final surface finish of 1 µm and carbon-coated prior to analysis. SEM observations were performed using a FEI Inspect S50 microscope (FEI, Hillsboro, OR, USA) operated in high-vacuum mode at an acceleration voltage of 15 kV and a beam current intensity of 50 nA. Elemental mapping was carried out using a Bruker X-Flash SDD detector (10 mm^2^ active area).

#### 2.4.7. Porosity

Total porosity accessible to water of 28 d-old WBC pastes was determined following NF P 18-459 (2010). Three cylindrical paste samples (≈15 g per sample) were placed in a vacuum desiccator at 25 mbar for 4 h. Then, samples were covered with demineralized water while maintaining the vacuum pressure at 25 mbar for a minimum of 44 h. After the immersion period, suspended and saturated weights of samples were determined. Finally, samples were dried to a constant weight in an oven at 75 °C to preserve the integrity of brushite, which is unstable at higher temperatures [38,45]. Pore size distribution was determined using Mercury Intrusion Porosimetry (MIP) with an AutoPore IV 9500 Porosimeter from Micromeritics Instrument Corporation (Norcross, GA, USA), following the ISO 15901-1 (2005) standard [46]. MIP analyses were performed on small pieces of paste of approximately 1–3 g, after hydration stoppage and drying, as described in Section 2.4.1. The mercury pressure varied from 0.0014 MPa to 400 MPa, and the pore entry size diameters were >3 nm.

## 3. Results and Discussion

### 3.1. Characterization of Pristine WBC Paste Samples

**Figure 2:**
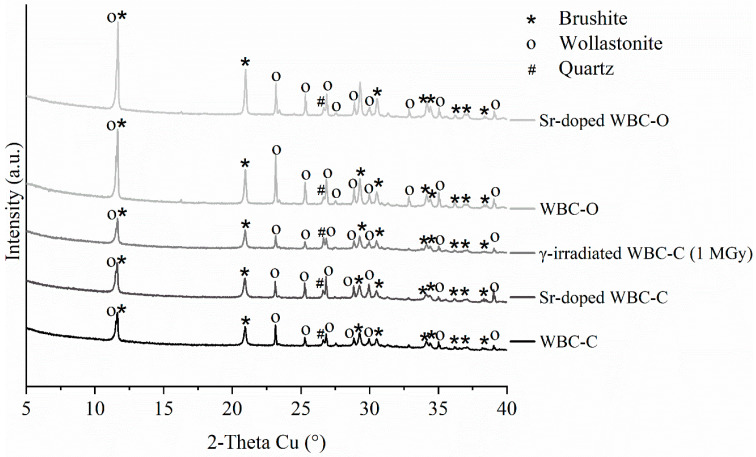
X-ray diffraction patterns of WBC-C, WBC-O, Sr-doped WBC-C, Sr-doped WBC-O, and γ-irradiated (1 MGy) WBC-C pastes.

The pristine WBC-C and WBC-O paste samples exhibited very similar diffraction patterns after 28 days of curing under endogenous conditions (Figure 2). Both materials contained brushite (CaHPO_4_·2H_2_O) and residual wollastonite (CaSiO_3_). Quartz, an impurity present in the wollastonite powder, was also evidenced. The corresponding Sr-doped pastes (Sr-doped WBC-C and Sr-doped WBC-O) displayed identical mineralogical features. No new crystalline phase related to the addition of strontium in the cement matrices was detected.

The weight fractions of each crystalline phase and of the total amorphous were determined by Rietveld refinement. The results are presented hereafter for reference for WBC-C and WBC-O pastes, but they were very similar to Sr-doped samples due to their very low Sr content (0.09% of Sr(NO_3_)_2_). To calculate the mass of each phase at 28 d, it was first necessary to determine the mass of the solid fraction. This latter could not be simply obtained by adding the bound water content determined by TGA to the initial mass of cement, since the reactions occurring during hydration not only consumed water, but also the ions from the mixing solution (phosphate, aluminum, zinc, boron, and sodium). A previous study showed that the pore solution contained very low concentrations of silicate (less than 2 mmol·L^−1^), regardless of the characterization time at early age [18]. In addition, the pore solution pH of WBC-based materials evolves from values below 1 to approximately 5–6 during hydration [47]. Within this pH range, the solubility of amorphous silica, which is the most soluble Si-containing cement hydrate, varies only slightly and remains close to 2 mmol·L^−1^ [48]. Therefore, as a first approximation, dissolved silica was neglected, and silicon was considered to be present only in the solid phase, as amorphous silica, wollastonite, and quartz. According to the mass conservation equation (Equation (6)), the total mass of silicon in the solid phase at 28 d ($m_{Si}^{solid}$) was thus assumed to be equal to the initial mass of silicon introduced via wollastonite and quartz:

(6) $$m_{Si}^{solid}\;\approx \;m_{Si}^{init}\;$$

The mass of the solid at 28 d ($m^{solid}$) was then derived from Equation (7):

(7) $$m^{solid}=\frac{m_{Si}^{solid}}{f_{Si}^{solid}}\approx \frac{m_{Si}^{init}}{f_{Si}^{solid}}$$

with $f_{Si}^{solid}$ as the weight fraction of Si in the solid.

The latter was determined by XRF (Table 2). Then, knowing the mass of the solid at 28 d, the mass of each crystalline phase was calculated using the Rietveld refinement results. The mass of amorphous silica ${(m}_{SiO_{2}\cdot 3.93H_{2}O})\;$ was derived from the mass balance equation (Equation (8)), considering SiO_2_·3.93H_2_O stoichiometry established experimentally by Jdaini [49] in a 28 d-old WBC paste prepared with a mixing solution containing H_3_PO_4_ (9 mol·L^−1^) and borax (0.05 mol·L^−1^). Assuming uncertainties of 10% for the Rietveld phase quantification and XRF analysis, 5% for the fraction of Si in the solid phase (i.e., considering that 95% of the initial Si mass is present in the solid instead of 100%), and 0.05% for weighing operations, uncertainty propagation yielded an estimated ~15% uncertainty in the mass of crystalline phases. Finally, the Ca/P, Zn/P, and Al/P molar ratios in the amorphous phase were calculated using mass conservation (Table 2):

(8) $$m_{SiO_{2}\cdot 3.93H_{2}O}=\left(m_{SiO_{2}}^{init}-m_{CaSiO_{3}}\times \frac{M_{SiO_{2}}}{M_{CaSiO_{3}}}\right)\left(1+3.93\times \frac{M_{H_{2}O}}{M_{SiO_{2}}}\right)$$

with $m_{CaSiO_{3}}$ as the mass of wollastonite at 28 d, and $M_{SiO_{2}}$, $M_{CaSiO_{3}}$ and $M_{H_{2}O}$ as the molar weights of silica, wollastonite, and water, respectively.

The concentration of boron in the solid fraction of the cement paste could not be determined since lithium metaborate was used as a reagent for alkaline fusion. Its contribution to the mass balance was neglected in the calculations. However, it is worth noting that the mass fraction of boron in the cement pastes remained very low (0.54% for WBC-C and 0.18% for WBC-O).

Although referred to as “brushite” cements, both pristine WBC-C and WBC-O paste samples were primarily composed of amorphous phases (aluminophosphate and hydrated silica). Brushite represented only 6.6 ± 1.6 wt.% of the 28 d-old WBC-C (including the pore solution) and 18.5 ± 4.5 wt.% of the 28 d-old WBC-O. The lower brushite content in WBC-C was consistent with the presence of zinc in the formulation, which is known to inhibit brushite formation [47]. The Zn/P and Al/P molar ratios of the aluminophosphate phase in WBC-C were consistent with those determined by Laniesse [47] on a similar material by SEM/EDX analyses (Zn/P = 0.15 ± 0.02, Al/P = 0.20 ± 0.02). A discrepancy was noticed concerning the Ca/P ratio (1.1 ± 0.1 in this study vs. 0.7 ± 0.05 in [47]). Nevertheless, Laniesse reported Ca/P values of 0.9 ± 0.1 for paste samples aged of 90 d, 180 d, and 360 d, which were in better agreement with our findings.

WBC-O contained a higher amount of unreacted (residual) wollastonite (9.8 ± 2.4 wt.%) than WBC-C (3.8 ± 0.9 wt.%). This trend was also reported by Laniesse [47], who observed that increasing the initial aluminum concentration in the mixing solution resulted in a higher residual wollastonite content at 28 days. This effect can be attributed to the preparation protocol: aluminum is introduced as a metallic powder, which reacts with protons (Al + 3 H^+^ → Al^3+^ + 3/2 H_2_) in the mixing solution. As more aluminum is added, the acidity decreases, which, in turn, slows down the dissolution of wollastonite.

Despite its lower initial aluminum content, WBC-C contained a larger fraction of amorphous aluminophosphate than WBC-O. This finding, also reported by Laniesse [47], may be related to the presence of zinc in WBC-C, which is absent in WBC-O. As previously discussed, Zn^2+^ is known to inhibit brushite crystallization [47], potentially promoting the formation of amorphous aluminophosphate instead. The Ca/P and Al/P molar ratios of the aluminophosphate phase in WBC-O were 0.84 ± 0.1 and 0.42 ± 0.1, respectively, which is consistent with values reported by Laniesse for similar WBC formulations [47].

**Figure 3:**
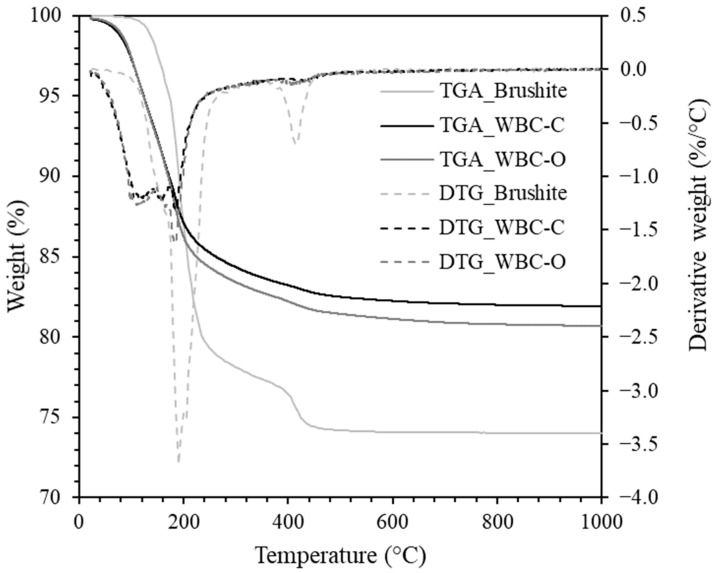
Thermograms of pristine WBC-C and WBC-O cement pastes and of pure brushite.

The thermograms and first derivatives of the pristine 28 d-old WBC-C and WBC-O paste samples are presented in Figure 3. For both materials, mass losses mainly occurred in three temperature ranges. In the 30–170 °C range, several thermal processes overlapped, including the evaporation of residual isopropanol (used for hydration stoppage) and physisorbed water, as well as the dehydration of amorphous silica (which occurs in the 45–100 °C and 135–150 °C intervals [50]) and aluminophosphate [18]. The weight loss observed between 170 and 220 °C was mainly attributed to brushite dehydration [38,45]. A further weight loss between 400 °C and 470 °C resulted from the dehydroxylation and condensation of monetite (CaHPO_4_) to form calcium pyrophosphate (Ca_2_P_2_O_7_) [38]. Because this process did not overlap with other decomposition reactions, it was used to estimate the brushite content in the solid fraction of WBC-C and WBC-O pastes. The calculated values (12.8 ± 2.0 wt.% for WBC-C and 20.7 ± 2.7 wt.% for WBC-O) were in reasonable agreement with those derived from the Rietveld refinement (8.2 ± 2.1 wt.% for WBC-C and 22.3 ± 5.6 wt.% for WBC-O). Both methods (TGA and XRD) consistently indicate a higher brushite content in the zinc-free WBC-O cement paste compared to WBC-C.

**Figure 4:**
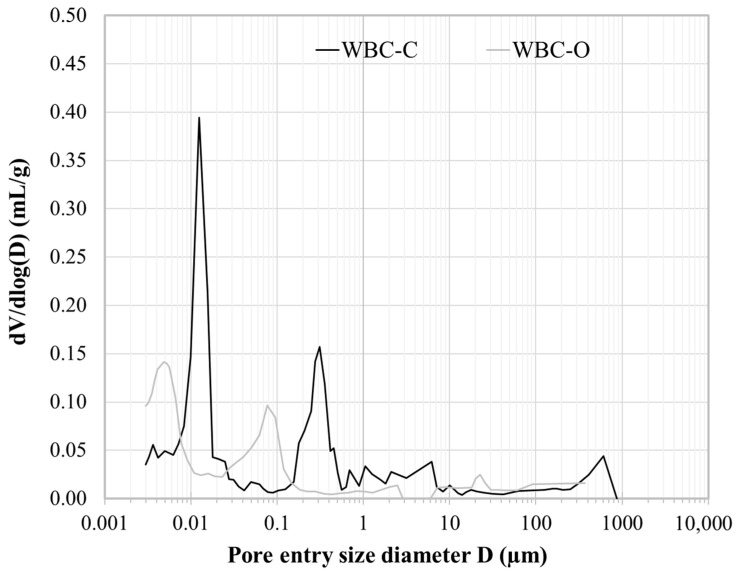
Pore entry size distributions of 28 d-old pristine WBC-C and WBC-O pastes obtained by MIP.

After 28 d of curing, WBC-C paste exhibited a total porosity accessible to water of 27.3 ± 0.7%, in good agreement with the total porosity accessible to mercury (28%). For comparison, Frizon et al. [36] reported a similar total porosity of 27% for a Portland cement paste with a water-to-cement (w/c) ratio of 0.35. The distribution of pore entry diameters (Figure 4) revealed that WBC-C paste presented two main modes centered at 0.012 µm and 0.3 µm, as well as a few macropores (mode at 600 µm) due to air entrapment, and also to the decarbonation of wollastonite in contact with the acidic mixing solution, which released carbon dioxide. In contrast, the WBC-O paste displayed its main pore size modes at finer diameters, around 0.005 µm and 0.08 µm. This refinement is consistent with the expected influence of aluminum on the pore structure. Laniesse et al. [18] showed that aluminum strongly influences the average diameter of mesopores: the higher its concentration, the smaller the pores. The average mesopore diameter of WBC-C (~107 Å at 90 d and ~80 Å at 180 d) was much larger than the values reported for WBC-O cement paste (~40 Å at 28 days) [18].

The porosity of WBC-C paste was less refined than that of a Portland cement paste at w/c = 0.35, where the peaks corresponding to capillary porosity and hydrate porosity were centered at 0.15 µm and 0.003 µm respectively [36].

**Figure 5:**
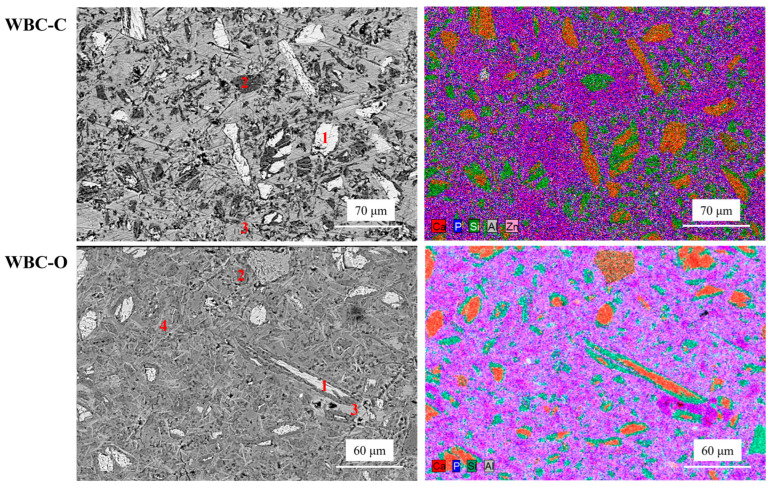
SEM backscattered electron images and elemental mapping of 28 d-old WBC-C (**top**) and WBC-O (**bottom**) pastes (1: wollastonite, 2: silica, 3: brushite, and 4: aluminophosphate).

SEM imaging was also performed to compare the microstructure of 28 d old WBC-C and WBC-O pastes (Figure 5). The WBC-C paste exhibited a more heterogeneous microstructure, with high-density regions corresponding to residual wollastonite and brushite, and lower-density microporous zones mainly composed of silica. In contrast, in the WBC-O paste, brushite (corresponding to bright purple zones on the elemental maps) seemed to be more homogeneously dispersed and surrounded by the aluminophosphate phase (indicated by light purple). In both samples, silica was observed to replace fully or partially dissolved wollastonite grains while preserving their original morphology, suggesting incongruent dissolution of wollastonite as previously reported [51].

### 3.2. Gamma Irradiation of WBC-C Paste

#### 3.2.1. Hydrogen Gas Production

**Figure 6:**
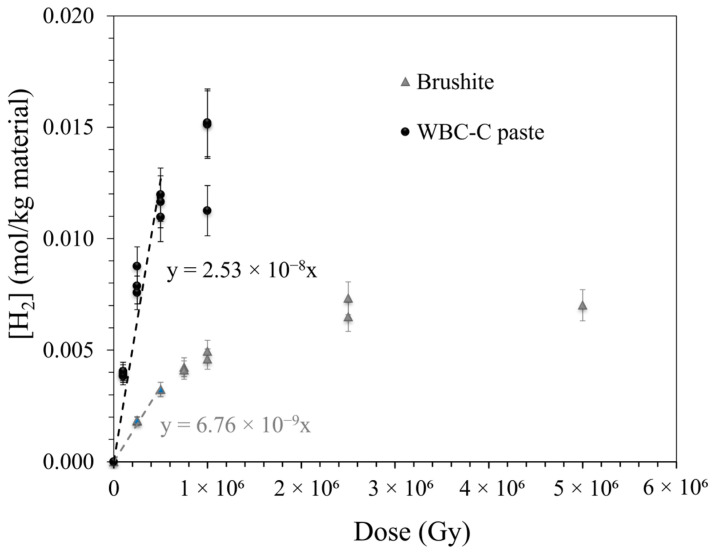
H_2_ production by WBC-C paste and brushite as a function of the integrated dose (γ-irradiation).

28 d-old WBC-C paste samples, as well as brushite, their main crystalline hydrate, were submitted to external gamma irradiation. Figure 6 plots the concentration of hydrogen gas released in the glass ampoules, normalized with respect to the weight of material, as a function of the integrated dose. In both cases, this concentration increased almost linearly up to 500 kGy, but tended to level off at higher doses, likely due to a recycling effect [27].

The radiolytic yields, *G*(*H*_2_)*_material_*, were given by the slopes of the regression lines in the 0–500 kGy dose range (Equation (3)) (Table 3 and Table 4). The yields were also normalized following (Equation (4)) with respect to the total mass of water present in the materials, including both evaporable and non-evaporable water.

The H_2_ radiolytic yields measured for WBC paste were of the same order of magnitude as those reported for geopolymer [52], Portland [29,53], or calcium sulfoaluminate [29] cement pastes. In comparison, calcium aluminate and magnesium phosphate cement pastes had significantly lower yields [30,34].

WBC paste constitutes a complex medium owing to its high heterogeneity. The various potential sources of H_2_ (namely, the different forms of water, including free water present in the pore solution or adsorbed onto solid surfaces, and bound water within cement hydrates (i.e., structural crystallization water and hydroxyl groups)) make it challenging to disentangle their respective contributions, all the more since these contributions may be coupled. In Portland cement pastes, radiolytic H_2_ has long been assumed to arise predominantly from the decomposition of liquid water [27]. However, increasing evidence now suggests that the radiolysis of bound water also contributes to H_2_ production [29,30,35].

The apparent *G*(*H*_2_)*_water_* yield calculated in this work for WBC-C paste (1.10 ± 0.11 × 10^−7^ mol/J) was about three times higher than that reported for free water (0.44 ± 0.04 × 10^−7^ mol/J [54]) (Table 3). This behavior could originate from several factors. The pore solution, containing a large panel of dissolved species (Al, Ca, P, Zn, B and Na) with a pH close to 6 [18], has a more complex chemical composition than pure water. Confinement of water within the cement matrix could also promote recombination of reductive species, leading to H_2_ formation, and/or facilitate efficient energy transfer from the material to the neighboring -OH groups. Similar results were observed by Chupin for geopolymers [52], and were also reported in the case of C-S-H (Table 4) [55].

**Table 4:** Comparison of H_2_ radiolytic yields for different cement hydrates (γ-rays).

Cement Hydrate	Dose	*G*(*H*_2_)*_material_* × 10^−7^ (mol·J^−1^)	*G*(*H*_2_)*_water_* × 10^−7^ (mol·J^−1^)	Ref.
Brushite CaHPO_4_·2H_2_O	250 kGy up to 5 MGy	0.070 ± 0.007	0.26 ± 0.02	This work
Gibbsite Al(OH)_3_	200 kGy	0.009 ± 0.005	0.027 ± 0.003	[30]
Katoite Ca_3_Al_2_(OH)_12_	200 kGy	0.003 ± 0.002	0.011 ± 0.001	
Calcium monocarboaluminate hydrate Ca_4_Al_2_(CO_3_)(OH)_12_·5H_2_O	200 kGy	0.12 ± 0.01	0.38 ± 0.04	
Portlandite Ca(OH)_2_		0.21		[56]
	200 kGy	0.042 ± 0.004	0.19 ± 0.03	[57]
	150 kGy	0.081 ± 0.005	0.33 ± 0.02	[35]
Brucite Mg(OH)_2_	200 kGy	0.055 ± 0.006	0.18 ± 0.03	[57]
		0.053		[56]
Calcium silicate hydrate (C-S-H) with CaO/SiO_2_ of:	100 and 200 kGy			[55]
0.80		0.61 ± 0.06	3.23 ± 0.32	
0.97		0.58 ± 0.06	3.11 ± 0.31	
1.14		0.49 ± 0.05	2.85 ± 0.29	
1.30		0.42 ± 0.04	2.44 ± 0.24	
1.40		0.36 ± 0.04	2.13 ± 0.21	
Bulk water (pH 13)		0.44 ± 0.04		[54]

The H_2_ radiolytic yield of brushite (CaHPO_4_·2H_2_O) given in Table 4 should be considered as indicative. Indeed, two main uncertainties were associated with its determination: *(i)* The sample was not dried before irradiation. Thus, uncontrolled sorption of water could lead to overestimating the H_2_ production. *(ii)* The brushite sample purity was 95.8% only. Nevertheless, the *G*(*H*_2_) value was of the same order of magnitude as those reported for portlandite, brucite, and calcium monocarboaluminate hydrate (Table 4), but significantly higher than those reported for gibbsite and katoite, the two main hydrates of calcium aluminate cement that are known to exhibit good stability under irradiation [30].

If we assume, like Acher et al. [30], that the H_2_ production yield of WBC paste is the sum of the yields of its hydrates and of free pore water, brushite, which accounts for 6.6 wt.% of the paste sample, would only contribute to 1.85% of the H_2_ production. This suggests that water present in the amorphous hydrates and/or in the pore solution would be more prone to decompose into hydrogen gas under irradiation.

#### 3.2.2. Mineralogy of Irradiated Materials

**Figure 7:**
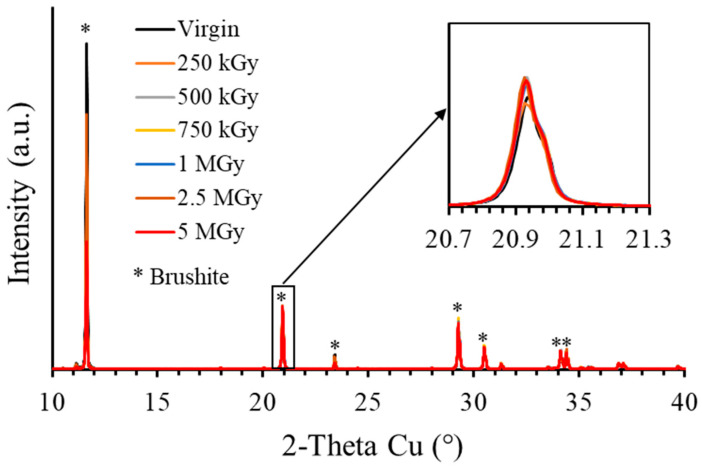
XRD patterns of virgin and γ-irradiated brushite samples.

**Figure 8:**
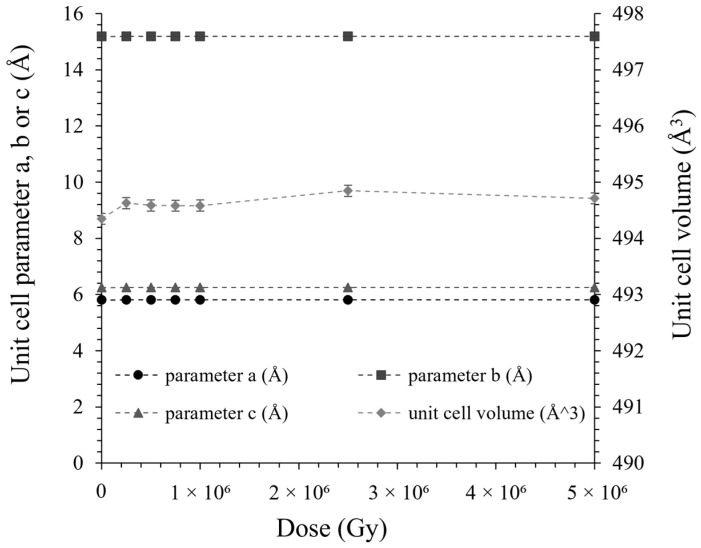
Evolution of unit cell parameters (a, b, and c) and volume of virgin and γ-irradiated brushite samples as a function of the irradiation dose.

The mineralogy of WBC-C paste was characterized by XRD after irradiation at a dose of 1 MGy (Figure 2). Its X-ray diffraction pattern was very similar to that of the virgin sample. Both materials contained brushite, residual wollastonite, and quartz. No new crystalline or amorphous phase that could produce a halo in the XRD pattern was observed. Moreover, the diffraction peaks did not show any broadening that could indicate the creation of defects in the crystalline phases induced by gamma irradiation. This was confirmed by investigating brushite more specifically, the main crystalline hydrate, after irradiation at doses ranging from 250 kGy to 5 MGy (Figure 7). No peak broadening was observed, nor the formation of new phases under irradiation. The crystal cell parameters of brushite were obtained from Rietveld refinement of the XRD patterns and showed no significant evolution with the integrated dose (Figure 8). The maximum relative volume change, Δ*V*/*V* = +0.073%, achieved by the unit cell of brushite under gamma irradiation (~1.2 MeV) at a dose of 5 MGy was approximately 3.57 times lower than the maximum expansion experienced by brushite under electron irradiation (2.5 MeV) at a much higher dose of 270 MGy, Δ*V*/*V* = +0.246% [58]. Amorphization of brushite into amorphous calcium pyrophosphate, which was observed under electron irradiation at doses higher than 270 MGy [33], was not observed in this study: the crystalline structure of brushite remained stable under gamma irradiation up to 5 MGy.

**Figure 9:**
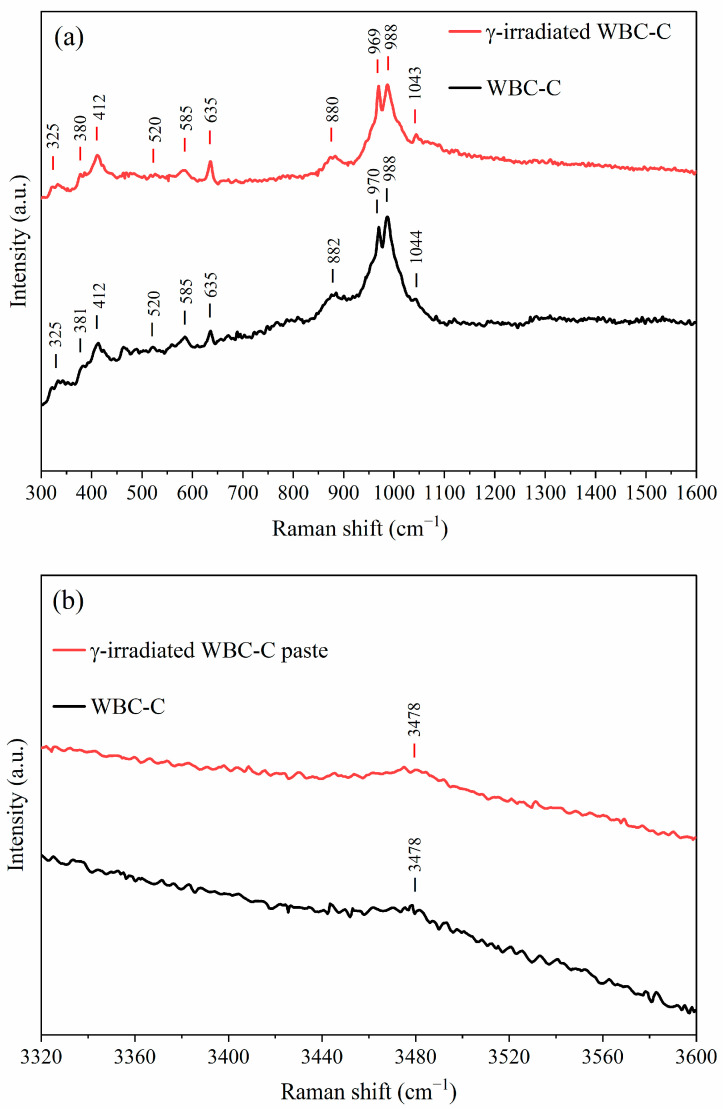
Raman spectra of pristine and γ-irradiated (1 MGy) WBC-C paste samples in the (**a**) 300–1600 cm^−1^ and (**b**) 3320–3600 cm^−1^ spectral regions.

The influence of g-irradiation on the local environment in the WBC paste was also investigated by Raman spectroscopy. Pristine and irradiated WBC paste samples exhibited very similar spectra (Figure 9) across the investigated regions (300–1600 cm^−1^, Figure 9a; 3320–3600 cm^−1^, Figure 9b). Band assignment remained challenging due to the absence of any reference data on the amorphous aluminophosphate phase, which likely contributed to the spectra. O–P–O bending modes of PO_4_^3−^ in brushite were observed at 330, 381, 412, and 585 cm^−1^, while the O–P–OH bending of HPO_4_^2−^ appeared near 520 cm^−1^ [59]. Wollastonite-related features were also present, including Si–O stretching bands at 635 cm^−1^ and ~970 cm^−1^, as well as a bending band at 585 cm^−1^ [60]. The most intense peak at 988 cm^−1^ corresponded to the symmetric P–O stretching of HPO_4_^2−^, with a weaker band at 1044 cm^−1^ assigned to asymmetric P–O stretching and a band at 882 cm^−1^ reflecting symmetric P–OH stretching [61,62]. In the high-frequency region (3320–3600 cm^−1^), a broad component centered at ~3478 cm^−1^ was observed, attributed to O–H stretching of water molecules [62]. Collectively, these Raman results show that WBC paste exhibits good stability under gamma irradiation in a range of doses relevant for LL- IL waste conditioning.

### 3.3. Leaching of Sr-Doped WBC Pastes

#### 3.3.1. Characterization of the Leachates

**Figure 10:**
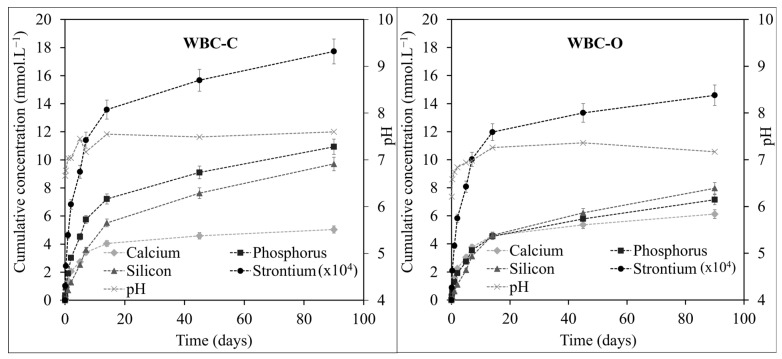
Evolution with time of pH and cumulative concentrations of Ca, P, Si, and Sr ([Sr] × 10^4^) released in the leachates for WBC-C (data from [24]) (**left**) and WBC-O (**right**) paste samples.

Leaching of Sr-doped WBC-C and WBC-O paste samples led to the release of Ca, Si, P, and Sr into solution. This release was rapid in the first few days, and then gradually slowed down (Figure 10). Overall, slightly lower concentrations were measured for WBC-O compared to WBC-C, suggesting improved element retention in the optimized formulation. For both materials, the leached concentration of Sr, always below 20 ppb, was more than three orders of magnitude lower than those of Ca, Si, and P. The Al and Zn concentrations always remained below the detection limit, indicating that the amorphous aluminophosphate phase exhibited better chemical durability than brushite under the investigated leaching conditions. The pH of the leachates, which was measured after each renewal, showed limited change over the test period, remaining within the range 6.5–7.5 (Figure 10). Using the Chess geochemical speciation software and the associated thermodynamic database [63], the saturation indices of the leachates versus relevant mineral phases were calculated (see Tables S1 and S2 in the Supplementary Information). A positive saturation index indicates oversaturation, whereas a negative value reflects undersaturation of the solution with respect to the corresponding solid phase. For both materials, the leachates were undersaturated with respect to SrHPO_4_, Sr(OH)_2_, SrSiO_3_, and brushite, but rapidly became oversaturated with respect to calcium hydroxyapatite. However, no turbidity or solid precipitation was visually observed in the leachates throughout the study.

**Figure 11:**
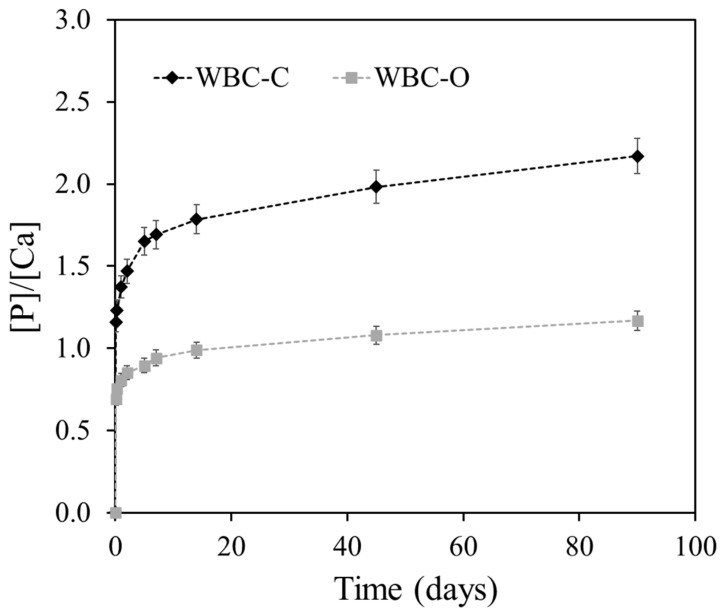
Evolution of the [P]/[Ca] concentration ratio in the leachates with time for Sr-doped WBC-C and Sr-doped WBC-O paste samples.

The evolution of the [P]/[Ca] concentration ratio in the leachates reveals distinct behaviors between the two cement pastes (Figure 11). For WBC-O, the ratio remained close to 1 throughout the test, suggesting a congruent dissolution of brushite (CaHPO_4_·2H_2_O). In contrast, WBC-C exhibited a progressive increase in the [P]/[Ca] ratio, which reached values close to 2 by the end of the test. This trend aligns with previous XRD results reported for the same material [24], which showed the precipitation of calcium-deficient hydroxyapatite (CDHA), Ca_9_(HPO_4_)(PO_4_)_5_(OH), on the exposed surface of the leached WBC-C paste sample.

**Figure 12:**
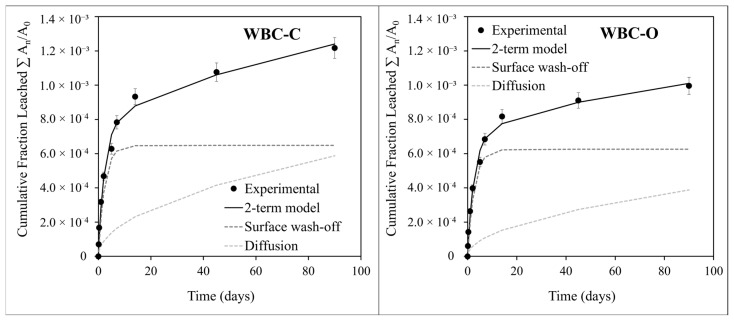
Experimental and calculated cumulative fractions of strontium leached from Sr-doped WBC-C (data from [24]) (**left**) and Sr-doped WBC (**right**) pastes. Use of a 2-term model, taking into account surface wash-off and diffusion in the pore solution.

The cumulative fractions of strontium leached (CFL) from both Sr-doped WBC pastes over 90 d are presented in Figure 12. At the end of the test, CFL values reached (1.22 ± 0.06) × 10^−3^ for WBC-C and (9.96 ± 0.49) × 10^−4^ for WBC-O, corresponding to the leaching of approximately 0.12% and 0.10% of the initial Sr content in the paste samples, respectively. These results indicate slightly improved Sr retention in WBC-O compared to WBC-C. The obtained CFL values are more than one order of magnitude lower than the data reported for Portland cement paste, which exhibits a CFL of around 5 × 10^−2^ at w/c = 0.5 [64].

#### 3.3.2. Modeling the Leaching of Strontium from WBC Pastes: Evaluation of the Diffusion Coefficient and Leachability Index

According to the literature, leaching from cementitious materials typically occurs via three main processes: surface exchange phenomena, diffusion of dissolved species in the pore network, and matrix dissolution [65,66]. Côtê et al. [65] proposed a semi-empirical kinetic model that accounts for these mechanisms, incorporating contributions from surface wash-off (representing the “initial fraction leached”), diffusion, and dissolution. The cumulative fraction leached (CFL) is described as a function of time using equation (Equation (9)):

(9) $$CFL\;\left(t\right)=\frac{\sum A_{n}}{A_{0}}=\;k_{1}\left(1-e^{-k_{2}t}\right)+\;k_{3}t^{1/2}+\;k_{4}t$$

To identify the dominant mechanisms controlling strontium leaching from WBC pastes, regression analysis was performed on the experimental CFL data using Equation (9). Note that several assumptions were made to derive the leaching models leading to Equation (9):

1. Time-invariant chemical environment: The leaching solution was periodically renewed to prevent accumulation of released species (the Ca, P, and Si concentrations remained below 1 mmol·L^−1^ (Ca) and 2 mmol·L^−1^ (P, Si), whereas the Sr concentration did not exceed 0.3 µmol/L between two renewals), and the pH remained relatively stable throughout the test (comprised between 6.5 and 7.5).
2. Semi-infinite solid geometry: As defined by the ANS/ANSI 16.1 specification, the semi-infinite assumption is valid if the cumulative fraction leached is less than 20%. In this study, the cumulative fraction of strontium leached remained well below this threshold (~0.12% for WBC-C and 0.10% for WBC-O at the end of the test).
3. Zero surface concentration of strontium: The Sr concentrations in the leachates were consistently low (<20 ppb), and the frequency of leachant renewal was high enough to support the boundary condition of zero surface concentration.

As a first approach, these assumptions were thus considered reasonably satisfied.

The optimized values of the model parameters are presented in Table 5 and Table 6 for Sr-doped WBC-C and Sr-doped WBC-O pastes, respectively. In both cases, the model showed excellent agreement with the experimental data, with adjusted r^2^ values of 0.992 for WBC-C and 0.991 for WBC-O. Notably, parameter k_4_ associated with matrix dissolution was not statistically different from zero at a 95% confidence level for either material. This means that leaching was governed primarily by surface wash-off and diffusion. As a result, the model was simplified by omitting the third term in Equation (9), and parameters *k*_1_, *k*_2_, and *k*_3_ were recalculated. Figure 12 compares the regression lines with the experimental CFL data for both paste samples. As expected, surface wash-off dominated during the initial phase (day 1), followed by diffusion-controlled leaching over the remainder of the test.

The apparent diffusion coefficient *D_a_* was derived from constant *k*_3_ (Equation (10)) [65]:

(10) $$D_{a}=\pi {\left(\frac{k_{3}\;V}{2\;S}\right)}^{2}$$

where *S*/*V* is the geometrical surface area-to-volume ratio of the specimen.

The WBC-O paste sample exhibited a lower Dₐ value ((2.1 ± 1.2) × 10^−15^ cm^2^·s^−1^) compared to WBC-C ((4.8 ± 1.6) × 10^−15^ cm^2^·s^−1^) [24], indicating enhanced Sr retention in the optimized formulation. The improved performance is likely related to differences in the mineralogical assemblages of the two cement pastes, which result in distinct microstructural properties. As reported by Laniesse et al. [18], increasing the initial aluminum concentration in the mixing solution from 1.6 mol·L^−1^ in WBC-C to 2.5 mol·L^−1^ in WBC-O produces a denser matrix with finer mesoporosity. This refined microstructure may contribute to slowing down the diffusion of Sr, thereby improving its retention in WBC-O.

**Figure 13:**
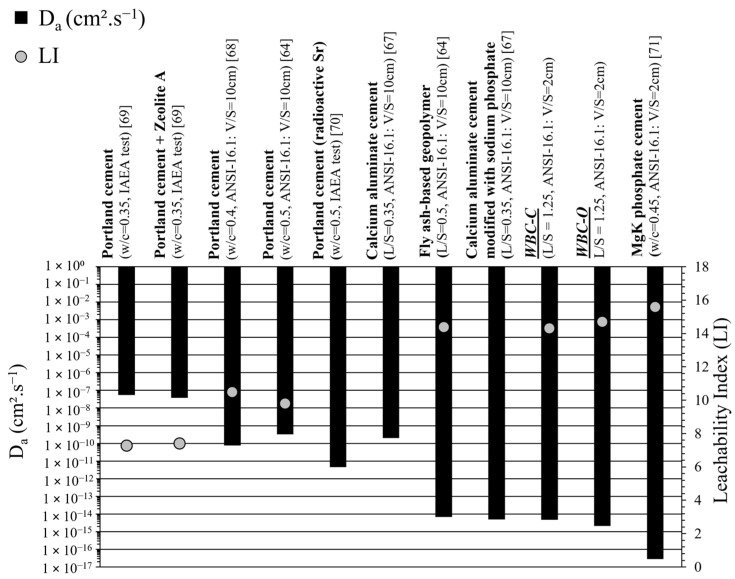
Comparison of D_a_ values and LI of strontium reported for different materials.

Figure 13 presents a tentative comparison between the apparent diffusion coefficients measured in this study and those reported in the literature for various binder systems. Given the diversity of experimental protocols and testing conditions, the reported values span a wide range and should be compared with caution.

The materials investigated in this work fall within the group exhibiting low Sr diffusivity, with retention performance comparable to that of sodium polyphosphate-modified calcium aluminate cement [67] or fly ash-based geopolymers [64], and it is markedly improved relative to Portland cement [64,68,69,70]. The lowest diffusivity values, however, have been reported for magnesium potassium phosphate-based matrices [71].

According to the ANSI specification, the leachability index (*LI*), defined by Equation (11), can be used to evaluate the efficiency of radionuclide immobilization in the matrix:

(11) $$LI=log\left(\frac{\beta }{D_{a}}\right)$$

where *β* is a reference constant equal to 1 cm^2^·s^−1^.

The calculated *LI* values for the WBC pastes (*LI* = 14.3 ± 0.2 for WBC-C and *LI* = 14.7 ± 0.2 for WBC-O) largely exceeded the minimum threshold (*LI* = 6) recommended by the US Nuclear Regulatory Commission for radioactive waste disposal.

## 4. Conclusions

This study evaluated the potential of wollastonite-based brushite cements (WBCs) for the conditioning of low- or intermediate-level radioactive wastes contaminated with strontium-90. Two formulations were investigated: a commercial reference (WBC-C) and an optimized binder (WBC-O). After 28 days of curing under endogenous conditions, both pastes primarily consisted of amorphous phases, including hydrated silica and an aluminophosphate phase, along with crystalline brushite, residual wollastonite, and quartz.

The stability of WBC-C paste was first investigated under γ-irradiation. Up to a cumulative dose of 1 MGy, irradiation primarily induced water radiolysis, with dihydrogen production rates comparable to those reported for Portland cement-based and geopolymer matrices. Complementary experiments on brushite, the main crystalline hydrate in WBC paste, confirmed its structural stability up to 5 MGy and indicated a radiolytic yield of H_2_ close to that obtained for portlandite (Ca(OH)_2_), brucite (Mg(OH)_2_), and calcium monocarboaluminate (Ca_4_Al_2_(CO_3_)(OH)_12_·5H_2_O). A similar irradiation study remains to be performed on WBC-O paste.

The influence of paste mineralogy on Sr retention was also explored. WBC-O, formulated with a higher initial aluminum concentration (2.5 mol·L^−1^ vs. 1.6 mol·L^−1^ in WBC-C), showed improved performance, with a diffusion coefficient nearly half that of WBC-C. This enhancement may be attributed, at least partly, to finer mesoporosity resulting from higher aluminum content, which may slow down diffusion of strontium in the pore network. The leaching of strontium from both WBC pastes was well described using the semi-empirical model of Côté and Constable [65]. Two dominant mechanisms were pointed out: surface wash-off (early stage) and diffusion (later stage).

Overall, these results suggest that WBCs may represent a promising option for the immobilization of Sr-containing radioactive waste. Future research should consider assessing their long-term leaching performance under repository-relevant alkaline conditions, as concrete in repository environments may generate high-pH plumes upon resaturation, and further elucidating the mechanisms controlling strontium retention within WBC matrices.

## Figures and Tables

**Table 1:** Formulation of the investigated WBC pastes.

Sample Code	Composition of the Mixing Solution	[Sr^2+^] in the Mixing Solution (mg·L^−1^)	Mixing Solution (g)	Wollastonite (g)
WBC-C	[H_3_PO_4_] = 9.3 mol·L^−1^ [Al^3+^] = 1.6 mol·L^−1^ [Zn^2+^] = 1.5 mol·L^−1^ [B] = 0.6 mol·L^−1^	-	62.5	50
WBC-O	[H_3_PO_4_] = 9 mol·L^−1^ [Al^3+^] = 2.5 mol·L^−1^ [B] = 0.2 mol·L^−1^	-	62.5	50
Sr-doped WBC-C	[H_3_PO_4_] = 9.3 mol·L^−1^ [Al^3+^] = 1.6 mol·L^−1^ [Zn^2+^] = 1.5 mol·L^−1^ [B] = 0.6 mol·L^−1^	1000	62.5	50
Sr-doped WBC-O	[H_3_PO_4_] = 9 mol·L^−1^ [Al^3+^] = 2.5 mol·L^−1^ [B] = 0.2 mol·L^−1^	1000	62.5	50

**Table 2:** Chemical and mineralogical compositions of pristine 28 d-old WBC-C and WBC-O cement pastes.

XRF Analysis	WBC-C (wt.%)	WBC-O (wt.%)		Phase Composition	WBC-C (g/100 g of Wollastonite)	WBC-O (g/100 g of Wollastonite)
Ca	17.9	17.2		Brushite CaHPO_4_·2H_2_O	14.9 ± 2.2	41.2 ± 6.1
Si	13.4	13.3		Wollastonite CaSiO_3_	8.5 ± 1.2	22.1 ± 3.3
P	11.6	11.3		Quartz SiO_2_	2.5 ± 0.4	3.6 ± 0.6
Zn	3.69	-		Amorphous silica SiO_2_·3.93H_2_O	97.9 ± 14.6	81.8 ± 12.2
Al	1.4	2.65		Amorphous aluminophosphate	56.9 ± 8.5 Ca/P = 1.1 ± 0.1 Al/P = 0.16 ± 0.02 Zn/P = 0.17 ± 0.02	36.1 ± 5.4 Ca/P = 0.84 ± 0.1 Al/P = 0.42 ± 0.1 -
Na	0.902	-		Pore solution	44.4 ± 6.6	39.7 ± 5.9
Mg	0.392	0.352				
Fe	0.214	0.189				
				
**Mineralogical composition of pristine 28°d-old WBC-C and WBC-O pastes (wt.%)**						
**WBC-C**				**WBC-O**		
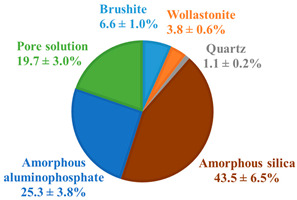				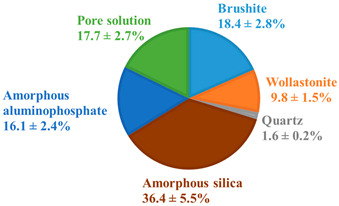		

**Table 3:** Comparison of H_2_ radiolytic yields for different cement pastes (γ-rays).

Type of Paste	Mass Fraction of Water (%)	Dose	*G*(*H*_2_)*_material_* × 10^−7^ (mol·J^−1^)	*G*(*H*_2_)*_water_* × 10^−7^ (mol·J^−1^)	Ref.
WBC-C	23.0	100 kGy up to 1 MGy	0.25 ± 0.02	1.10 ± 0.11	This work
Geopolymer Geo Na	33.0	100 and 250 kGy	0.13 ± 0.10	0.4 ± 0.04	[52]
Geo K	33.9		0.25 ± 0.02	0.75 ± 0.07	
Geo Cs	26.8		0.48 ± 0.04	1.80 ± 0.18	
Portland cement	16.7 37.5	1 MGy	0.13 ± 0.10 0.33 ± 0.03		[53]
Portland cement (CEM I 52.5 N SR0 CE PM-CP2 NF)	16.7	500 kGy	0.05 ± 0.01	0.31 ± 0.03	[30]
	23.1		0.07 ± 0.01	0.30 ± 0.04	
	28.6		0.10 ± 0.01	0.32 ± 0.03	
	33.3		0.11 ± 0.01	0.32 ± 0.03	
	37.5		0.11 ± 0.01	0.30 ± 0.03	
Calcium sulfoaluminate cement (85% clinker + 15% CaSO_4_)	28.6	100 and 200 kGy	0.10 ± 0.01	0.37 ± 0.01	
Calcium aluminate cement	16.6	500 kGy	0.009 ± 0.005	0.055 ± 0.005	[30]
	23.0		0.018 ± 0.002	0.078 ± 0.008	
	28.4		0.032 ± 0.003	0.11 ± 0.01	
	33.1	150 and 300 kGy	0.057 ± 0.006	0.17 ± 0.01	
	37.3		0.083 ± 0.008	0.22 ± 0.02	
Magnesium phosphate cement (molar ratios) MgO/KH_2_PO_4_ = 1 H_2_O/MgO = 5	40.5	150 and 300 kGy	0.084 ± 0.010	0.21 ± 0.03	[34]
Bulk water (pH 13)				0.44 ± 0.04	[54]

**Table 5:** Coefficients from the regression analysis of CFL data for Sr-doped WBC-C paste [experimental data from 24].

Model		Value	Standard Error	*t*-Value	Prob > |t| (%)
${CFL\;\left(t\right)=k}_{1}\left(1-e^{-k_{2}t}\right)+\;k_{3}t^{1/2}+\;k_{4}t$ *r*^2^*_adjusted_* = 0.992	k_1_	4.04 × 10^−4^	1.17 × 10^−4^	3.45	*
	k_2_	4.92 × 10^−1^	1.25 × 10^−1^	3.93	**
	k_3_	1.59 × 10^−4^	4.50 × 10^−5^	3.54	*
	k_4_	−7.94 × 10^−6^	3.67 × 10^−6^	−2.16	7.40
${CFL\;\left(t\right)=k}_{1}\left(1-e^{-k_{2}t}\right)+\;k_{3}t^{1/2}$ *r*^2^*_adjusted_* = 0.988	k_1_	6.48 × 10^−4^	6.25 × 10^−5^	10.37	***
	k_2_	4.27 × 10^−1^	8.20 × 10^−2^	5.20	**
	k_3_	6.20 × 10^−5^	9.53 × 10^−6^	6.50	***

Probability: ***< 0.1%; ** 0.1–1%; and * 1–5%.

**Table 6:** Coefficients from the regression analysis of CFL data for Sr-doped WBC-O paste.

Model		Value	Standard Error	t-Value	Prob > |t| (%)
${CFL\;\left(t\right)=k}_{1}\left(1-e^{-k_{2}t}\right)+\;k_{3}t^{1/2}+\;k_{4}t$ *r*^2^*_adjusted_* = 0.991	k_1_	3.94 × 10^−4^	1.09 × 10^−4^	3.61	*
	k_2_	4.04 × 10^−1^	0.91 × 10^−1^	4.41	**
	k_3_	1.31 × 10^−4^	4.01 × 10^−5^	3.27	*
	k_4_	−7.29 × 10^−6^	3.22 × 10^−6^	−2.27	6.4
${CFL\;\left(t\right)=k}_{1}\left(1-e^{-k_{2}t}\right)+\;k_{3}t^{1/2}$ *r*^2^*_adjusted_* = 0.987	k_1_	6.26 × 10^−4^	6.01 × 10^−5^	10.40	***
	k_2_	3.69 × 10^−1^	0.66 × 10^−1^	5.59	***
	k_3_	4.09 × 10^−5^	8.89 × 10^−6^	4.60	**

Probability: ***< 0.1%; ** 0.1–1%; and * 1–5%.

## Data Availability

The original contributions presented in this study are included in the article/Supplementary Material. Further inquiries can be directed to the corresponding author.

## Supplementary Materials

The following supporting information can be downloaded at: https://www.mdpi.com/article/10.3390/ma19061136/s1, Supplementary Information S1: Estimation of the water saturation degree of the WBC-C paste at 28 d; Supplementary Information S2: Calculation of saturation indices in the leachates; Table S1: Leaching of WBC-C paste by demineralized water—Chemical composition of the leachates and saturation indices with respect to calcium phosphate and strontium-containing phases; Table S2: Leaching of WBC-O paste by demineralized water—Chemical composition of the leachates and saturation indices with respect to calcium phosphate and strontium-containing phases.
