# Supplementary material for: Potential of Wollastonite-Based Brushite Cement for the Conditioning of Radioactive Waste Contaminated by 90Sr
Source: Materials (Basel). 2026 Mar 14;19(6):1136. doi: 10.3390/ma19061136 (PMC13027612; doi:10.3390/ma19061136)
Supplement: Supplementary file 1 [file materials-19-01136-s001.zip › materials-4164369-supplementary.pdf]

Supplementary Information

S1. Estimation of the water saturation degree of the WBC-C paste at 28 d

The initial water content of the WBC-C paste was calculated from the mix design: 125 g of mixing solution was added to 100 g of wollastonite. The dry extract of the mixing solution, measured after drying at 105 °C to constant mass, was 63.2 ± 0.1 wt.%, corresponding to a water content of 36.8 ± 0.1 wt.%. This represents 46.0 ± 0.2 g of water per 125 g of mixing solution.

Thermogravimetric analysis (TGA) indicated that bound water accounts for 18 wt.% of the hydrated solid after 28 days (Figure 3). The mass of hydrated solid formed from 100 g of wollastonite was derived from the phase assemblage determined by Rietveld refinement and XRF analyses (Table 2), yielding 180.7 ± 27.1 g. The corresponding bound water content was therefore 32.5 ± 4.9 g.

The free water content was calculated as the difference between total and bound water contents, giving 13.5 ± 5.1 g.

The pore volume was estimated from the total sample volume (110.5 mL, calculated from 100 g of wollastonite, absolute density of 2.9 $g \cdot cm^{-3}$, and 125 g of mixing solution, with a density of 1.645 $g \cdot cm^{-3}$, neglecting entrapped air) and the water-accessible porosity (27.3 ± 0.7 vol.%). This yielded a pore volume of 30.2 ± 0.8 mL.

The resulting degree of water saturation of the porosity was 45 ± 18%.

S2: Calculation of saturation indices in the leachates

Table S1: Leaching of WBC-C paste by demineralized water—Chemical composition of the leachates and saturation indices with respect to calcium phosphate and strontium-containing phases.

Sampling time	[Ca]	[P]	[Si]	[Al]	[Zn]	[Sr]	pH	Saturation indices				
	mmol.L^{-1}					µmol.L^{-1}		HAp*	Brushite	$SrHPO_4$	$Sr(OH)_2$	$SrSiO_3$
2 h	0.30	0.35	0.09	< dl	< dl	0.10	6.7	-0.63	-14.27	-4.95	-21.25	-12.61
7 h	0.44	0.56	0.17	< dl	< dl	0.14	6.8	1.33	-13.86	-4.55	-20.90	-11.98
24 h	0.65	1.00	0.46	< dl	< dl	0.22	7.0	4.17	-13.35	-3.99	-20.20	-10.85
48 h	0.67	1.11	0.57	< dl	< dl	0.22	7.0	4.32	-13.30	-3.95	-20.20	-10.76
5 d	0.68	1.49	1.25	< dl	< dl	0.23	7.5	6.50	-13.08	-3.66	-19.38	-9.59
7 d	0.66	1.24	1.06	< dl	< dl	0.22	7.2	5.03	-13.22	-3.83	-19.94	-10.22
14 d	0.64	1.46	1.92	< dl	< dl	0.22	7.6	6.80	-13.09	-3.67	-19.21	-9.24
45 d	0.55	1.89	2.11	< dl	< dl	0.21	7.5	6.37	-13.09	-3.60	-19.36	-9.35
90 d	0.45	1.83	2.08	< dl	< dl	0.21	7.6	6.40	-13.16	-3.58	-19.15	-9.14

* HAp = calcium hydroxyapatite, and dl = detection limit.

Table S2: Leaching of WBC-O paste by demineralized water—Chemical composition of the leachates and saturation indices with respect to calcium phosphate and strontium-containing phases.

Sampling time	[Ca]	[P]	[Si]	[Al]	[Sr]	pH	Saturation indices				
	mmol.L^{-1}				µmol.L^{-1}		HAp*	Brushite	$SrHPO_4$	$Sr(OH)_2$	$SrSiO_3$
2 h	0.30	0.35	0.09	< dl	0.10	6.7	-0.63	-14.27	-4.95	-21.25	-12.61
7 h	0.44	0.56	0.17	< dl	0.14	6.8	1.33	-13.86	-4.55	-20.90	-11.98
24 h	0.65	1.00	0.46	< dl	0.22	7.0	4.17	-13.35	-3.99	-20.20	-10.85
48 h	0.67	1.11	0.57	< dl	0.22	7.0	4.32	-13.30	-3.95	-20.20	-10.76
5 d	0.68	1.49	1.25	< dl	0.23	7.5	6.50	-13.08	-3.66	-19.38	-9.59
7 d	0.66	1.24	1.06	< dl	0.22	7.2	5.03	-13.22	-3.83	-19.94	-10.22
14 d	0.64	1.46	1.92	< dl	0.22	7.6	6.80	-13.09	-3.67	-19.21	-9.24
45 d	0.55	1.89	2.11	< dl	0.21	7.5	6.37	-13.09	-3.60	-19.36	-9.35
90 d	0.45	1.83	2.08	< dl	0.21	7.6	6.40	-13.16	-3.58	-19.15	-9.14

* HAp = calcium hydroxyapatite, and dl = detection limit.
